# Effect of Acarbose on Long-Term Prognosis in Acute Coronary Syndromes Patients with Newly Diagnosed Impaired Glucose Tolerance

**DOI:** 10.1155/2016/1602083

**Published:** 2015-12-06

**Authors:** Peng Yun, Ai-ming Du, Xue-jun Chen, Jing-cheng Liu, Hu Xiao

**Affiliations:** ^1^Department of Internal Medicine, School of Clinical Medicine, Yangtze University, Jingzhou, Hubei 434000, China; ^2^Department of Cardiology, Central Hospital of Jingzhou City, Hubei 434001, China; ^3^Department of Endocrinology, The First Affiliated Hospital of Yangtze University, Jingzhou, Hubei 434000, China

## Abstract

*Objective*. To investigate the effect of acarbose therapy on the long-term prognosis of patients with acute coronary syndromes (ACS) complicating newly diagnosed impaired glucose tolerance (IGT). *Methodology*. 135 patients hospitalized for ACS who had been newly diagnosed with IGT were randomly assigned to acarbose group (150 mg/day, *n* = 67) or control group (no acarbose, *n* = 68). All cases in each group were given the same elementary treatment. Mean follow-up was 2.3 years. The incidence of major adverse cardiovascular event (MACE) and carotid intima-middle thickness (CIMT) were statistically analyzed. *Results*. During the mean follow-up of 2.3 years, the risk of recurrent MACE in acarbose group was decreased significantly compared with that in control group (26.67% versus 46.88%, *P* < 0.05); at the same time, thickening of the CIMT was significantly slower than the control group ((1.28 ± 0.42) mm versus (1.51 ± 0.64) mm, *P* < 0.05). *Conclusions*. Acarbose can effectively reduce the risk of MACE in ACS patients with newly diagnosed IGT, simultaneously retarding the progression of carotid intima-media thickness.

## 1. Introduction

As gradual steps into aging society and changes of lifestyle, the prevalence of impaired glycometabolism and coronary artery disease (CAD) increases rapidly in China. Impaired glucose tolerance (IGT) has been regarded as a prediabetic state in which postprandial blood glucose is between normal glucose tolerance and overt diabetes mellitus (DM). It is well known that IGT is an independent risk factor of cardiovascular events (CV) and cardiovascular-associated mortality [1]. Acarbose can effectively reduce postprandial blood glucose and the progression from IGT to Type 2 DM (T2DM). STOP-NIDDM study had proved that acarbose therapy reduced the risk of any CV by 49%, of an acute myocardial infarction (AMI) by 91%, and of developing hypertension by 34% in IGT patients [2]. Emerging evidence suggested a high prevalence of unrecognized IGT and/or DM in patients admitted to hospital with acute coronary syndrome (ACS) [3]. Furthermore, such dysglycaemia has been shown to be associated with an increase in cardiovascular mortality. Therefore, we have reason to postulate that acarbose treatment may reduce major adverse cardiovascular event (MACE) in patients with ACS complicating impaired glycometabolism. No related research has been reported so far. Thus, the goal of present study is to determine whether acarbose can reduce the risk of recurrent MACE in ACS patients with newly diagnosed IGT.

## 2. Research Design and Methods

### 2.1. Study Patients

From March 2010 to August 2013, we randomly selected 426 cases of patients who were hospitalized in our two hospitals due to ACS. ACS was diagnosed by the presence of acute ischemic symptoms lasting ⩾20 min within 48 h before admission to hospital and electrocardiographic changes consistent with ACS [4]. Acute myocardial infarction (AMI) was diagnosed when creatine kinase-MB levels increased to at least twice the upper limit of normal or when troponin T levels were >0.1 ng/mL. Patients without AMI were considered to have unstable angina pectoris (all cases confirmed by percutaneous or computed tomography coronary angiography). Exclusion criteria were as follows: (1) cardiogenic shock or pulmonary edema (Killip classification ⩾ II) at admission, (2) history of diabetes, (3) history of hepatic diseases or/and renal dysfunction (serum creatinine level >2 mg/dL), (4) severe gastrointestinal disease or malignant tumors, and (5) female patients given sex hormone replacement therapy. All cases underwent the standard 75 g oral glucose tolerance test (OGTT) two weeks after admission, and we used the WHO criteria to classify the OGTT results. IGT was defined as having a fasting plasma glucose (FPG) <6.1 mmol/L and a postprandial glucose level at 120 min after the glucose load (2 hPG) ⩾7.8 mmol/L but <11.1 mmol/L. 135 cases were newly diagnosed with IGT. The present study followed Helsinki principle which was reviewed and approved by the Ethics Committee; all patients and their families provided informed consent.

### 2.2. Methods

135 IGT patients were randomly allocated, using random numbers generated by a computer, into the following two groups: the control group (68 cases) and acarbose group (67 cases). Each group was given standard medical therapy of CAD (including nitrate medications, ACE-I/ARB, *β*-blockers, statins, and antiplatelet drugs). Acarbose group was given additional acarbose (Bayer Pharmaceutical Co., Germany, three times/day, 25 mg/time start, and gradually increasing the amount to 50 mg/time in 2 weeks) on the base of fundamental treatment. Carotid intima-media thickness (CIMT) was measured using Siemens SEQUOIA512 ultrasonography, taking the point under 1 cm of carotid sinus as detection point and accounting the average of the left and right CIMT as the results. The carotid IMT was measured at baseline, and follow-up of all subjects, ΔCIMT, indicated the changes in CIMT. All patients were guided to take diet and exercise therapy, and having outpatient clinic or telephone follow-up for 1.0–4.5 years, the mean follow-up was 2.3 years. Incidence of MACE (including fatal cardiovascular events, nonfatal reinfarction, new-onset angina, cerebral stroke, and severe heart failure) was recorded.

### 2.3. Statistical Analysis

Statistical analysis was performed using SPSS 13.0. Continuous variables were expressed as mean and standard deviation, and categorical variables were expressed as using numbers and percentages. Comparisons among the two groups were performed using Student's *t*-test and paired *t*-test for continuous variables and *χ* ^2^ test for categorical variables. Values for *P* less than 0.05 were accepted as statistically significant.

## 3. Results

### 3.1. Clinical Background

The clinical profile of the subjects was shown in Table 1. There was no significant difference in age, gender, profiles for traditional risk factors, and past medication history between the two groups (*P* > 0.05). Of the total of 135 cases in the average follow-up of 2.3 years, 11 patients dropped out during the study which was 8.15% of all subjects (7 patients in the acarbose group: 5 male and 2 female; 4 patients in the control group: 2 male and 2 female). The cause was severe abdominal distension and diarrhea for 6 cases in the acarbose group, or lost for 1 case in acarbose group and 4 cases in control group, respectively; the incidence of serious gastrointestinal adverse reactions between the two groups was statistically significant (*χ* ^2^ = 6.373, *P* = 0.012).

### 3.2. The Risk of Recurrent MACE

Recurrent MACE was observed in the 124 cases that completed the study.  Table 2 demonstrated the numbers of MACE among subjects in the mean 2.3-year follow-up. The incidence of total MACE in the acarbose group and control group was 26.67% and 46.88%, respectively; there was significant difference between the two groups (*χ* ^2^ = 5.420, *P* = 0.020). In total MACE, the death of 3 patients in acarbose group and 5 patients in control group was due to cardiovascular events, and the cardiovascular caused mortality between the two groups was of no significant difference (*χ* ^2^ = 0.406, *P* = 0.524). However, the incidence of the secondary end-point events (nonfatal reinfarction, new-onset angina, cerebral stroke, and severe heart failure) of the acarbose group was prominently lower than that of control group (21.67% versus 39.06%, *χ* ^2^ = 4.410, *P* = 0.036).

### 3.3. Laboratory and Ultrasonography Examination

Table 3 shows the level of biochemical indicators, CIMT, blood pressure, and BMI of the two groups before and after treatment. All the indexes were not significantly different between the two groups before treatment (*P* > 0.05). After treatment, 2 hPG, HbA1c, CIMT, and ΔCIMT of the acarbose group were significantly lower than control group (*t* value was 8.731, 6.198, 2.440, and 18.622, resp., *P* < 0.05 or 0.01), while FPG, TC, TG, LDL-C, systolic pressure, diastolic pressure, and BMI had no statistical significance between the two groups after treatment (*t* value was 1.528, 0.242, 1.102, 0.214, 1.201, 0.883, and 1.866, resp., *P* > 0.05).

## 4. Discussion

Postprandial hyperglycemia is a hallmark characteristic in individuals with IGT and early T2DM and has been established as a key pathophysiological component of the mechanism underlying the development of diabetic complications [5]. Fluctuations in glucose levels following a meal are strongly associated with micro- and macrovascular complications not only in patients with T2DM, but also in individuals with IGT [6]. The risk for developing CAD or other MACE was increased almost threefold in subjects with IGT compared to people with normal glucose tolerance [7]. Kataoka et al. [8] found that multibranch coronary artery lesion which was common in T2DM patients had already emerged in the IGT stage. Recent study indicated that IGT was an independent risk factor for AMI and simultaneously increased the risk of recurrent CV after AMI [9]. Furthermore, Kitada et al. [10] showed that postprandial blood glucose above 8.9 mmol/L would increase the risk of recurrent MACE nearly one-fold in patients with AMI. There is a high prevalence of unrecognized dysglycaemia in patients with ACS; the present study found that 31.69% of the patients with ACS were newly diagnosed IGT; it was similar to previous researches [3, 11]. Thus, early intervention to IGT in ACS patients with the aim of reducing recurrent MACE should be anticipated.

IGT is a prediabetic state; several management strategies have been proposed for this early stage of dysglycaemia, including lifestyle modification and pharmacotherapies (e.g., acarbose, metformin, and thiazolidinediones) [12–14]. Although lifestyle modification is a vital part of dysglycaemia management, it is often insufficient to maintain long-term glycaemic control. Given that acarbose has a relatively modest efficacy of blood glucose lowering and can be administered to patients with heart failure and mild to moderate renal insufficiency, acarbose is safer than other drugs mentioned above for glucose management in ACS patients, because management of glucose levels within a given range and with minimal risk of hypoglycemia is recommended for the treatment of hyperglycemia in patients with ACS [15]. In the present study, 2 hPG and HbA1c of the acarbose group significantly reduced compared with those of control group (*P* < 0.01); this result was not unexpected. The starting dose of acarbose was semiquantity and gradually increased to normal dose (50 mg/time, three times/day), so only 6 patients (8.96%) withdrew from the trial due to severe gastrointestinal side effects. The other patients were well tolerated and with no hypoglycemia.

Previous studies [2, 16] had suggested that acarbose was useful in reducing the risk of MACE in patients with IGT; its cardiovascular protective effect might be due to the reduction of postprandial hyperglycemia and glucose variability, increased insulin sensitivity, induction of moderate weight loss, restoration of endothelial function, and so forth [17]. In the above mechanisms, the improvement of vascular endothelial function is reasonably important. Endothelial dysfunction plays an important role in the development of atherosclerosis and predicts CV outcomes independent of conventional CV risk factors [18]. Although the mechanism by which postprandial hyperglycemia induces endothelial dysfunction is not fully understood, oxidative stress-mediated disruptions in nitric oxide homeostasis are implicated as key role [19]. Endothelium-derived nitric oxide (NO) is one of the most potent known endogenous vasodilators and it plays an important role in the control of coronary blood flow by regulating vascular tone. Kato et al. [20] found acarbose improved postprandial endothelial function by improvement of postprandial hyperglycemia in patients with newly diagnosed T2DM. This notion had been recently reinforced by the finding that 24 weeks of acarbose monotherapy in newly diagnosed patients with T2DM was associated with increased levels of both fasting and postprandial glucagon-like peptide 1 (GLP-1), NO levels, and nitric oxide synthase (NOS) activity [21]. Thus, acarbose seemed to favorably affect endothelial function in the coronary arteries and contributed to an improved long-term prognosis. The present study showed that acarbose could effectively reduce total risk of MACE in ACS patients with newly diagnosed IGT (*P* < 0.05). This cardiovascular benefit mostly owed to the reduction of secondary endpoint events (*P* < 0.05), while the decline of cardiovascular disease mortality was not statistically significant (*P* > 0.05), which might be related to not long enough follow-up time and not enough number of patients enrolled.

The absolute value and change of CIMT are both indirect indicators of coronary atherosclerosis and independent predictors of long-term CV [22, 23]. Previous study had confirmed that acarbose could slow the progression of CIMT in patients with IGT or T2DM [24, 25], and Koyasu et al. [26] found that acarbose also could retard CIMT thickness and plaque formation in CAD patients with newly diagnosed IGT. The present study showed that acarbose slowed the progression of CIMT in ACS patients with IGT, which also indirectly suggested that acarbose delayed the development of coronary atherosclerosis in patients with IGT. In the present study, BMI in acarbose group had a distinctly downtrend compared with the control group (*P* = 0.064); it suggested that moderate loss of weight may be one of the possible reasons for the cardiovascular benefit.

In summary, the present study indicates that acarbose can effectively and safely retard the CIMT thickness and reduce the risk of recurrent MACE in ACS patients with newly diagnosed IGT. Therefore, acarbose can improve the prognosis of these patients.

## Figures and Tables

**Table 1:** Baseline characteristics of study patients and frequencies of medication usage before admission.

	Control group (*n* = 68)	Acarbose group (*n* = 67)	*P* value
*Basic characteristic *			
Age, year	61.62 ± 4.58	62.24 ± 5.16	0.461
Male, *n* (%)	42 (61.76)	39 (58.21)	0.673
Smoking, *n* (%)	31 (45.59)	28 (41.79)	0.657
AMI, *n* (%)	43 (63.24)	46 (68.66)	0.506
Hypertension, *n* (%)	46 (67.65)	45 (67.16)	0.637
LVEF, %	52.13 ± 4.81	51.74 ± 5.25	0.653
Revascularization (PCI/CABG), *n* (%)	38 (55.88)	41 (61.19)	0.531
BMI, kg/m^2^	25.82 ± 2.45	26.05 ± 3.24	0.427
*Medications *			
*β*-blocker, *n* (%)	16 (23.53)	13 (19.40)	0.559
ACE-I/ARB, *n* (%)	47 (69.12)	45 (67.16)	0.808
CCB, *n* (%)	18 (26.47)	20 (29.85)	0.662
Statin, *n* (%)	63 (92.65)	61 (91.04)	0.734
Aspirin, *n* (%)	64 (94.12)	62 (92.54)	0.713

AMI: acute myocardial infarction; PCI: percutaneous coronary intervention; CABG: coronary artery bypass grafting; BMI: body mass index; ACE-I: angiotensin-converting enzyme inhibitor; ARB: angiotensin II receptor blocker; CCB: calcium channel blocker.

**Table 2:** MACE among the study patients in the mean 2.3-year follow-up (*n*, %).

	Control group (*n* = 64)	Acarbose group (*n* = 60)
Cardiovascular death, *n* (%)	5 (7.81)	3 (5.00)
Nonfatal reinfarction, *n* (%)	7 (10.94)	2 (3.33)
New-onset angina, *n* (%)	9 (14.06)	5 (8.33)
Cerebral stroke, *n* (%)	4 (6.25)	2 (3.33)
Severe heart failure, *n* (%)	5 (7.81)	4 (6.67)
Total MACE, *n* (%)	30 (46.88)	16 (26.67)

MACE: major adverse cardiovascular events.

**Table 3:** Biochemical indicator level, CIMT, BMI, and blood pressure of two groups between pre- and posttreatment (mean ± SD).

	Control group		Acarbose group	
	Pretreatment *n* = 68	Posttreatment *n* = 64	Pretreatment *n* = 67	Posttreatment *n* = 60
FPG, mmol/L	5.84 ± 0.33	5.95 ± 0.54	5.92 ± 0.42	5.78 ± 0.69
2 hPG, mmol/L	8.76 ± 0.49	9.46 ± 1.22	8.98 ± 0.54	7.64 ± 1.08^#,▲^
HbA1c, %	6.28 ± 0.23	6.36 ± 0.51	6.30 ± 0.28	5.92 ± 0.24^#,▲^
Systolic, mmHg	148.54 ± 8.63	140.27 ± 6.25^*∗*^	150.16 ± 10.38	138.82 ± 7.14^▲^
Diastolic, mmHg	93.26 ± 5.34	88.51 ± 4.62^*∗*^	94.13 ± 7.18	87.73 ± 5.18^▲^
TC, mmol/L	6.24 ± 1.03	5.06 ± 0.96^*∗*^	6.38 ± 1.35	5.03 ± 0.87^▲^
TG, mmol/L	2.36 ± 0.58	1.74 ± 0.52^*∗*^	2.45 ± 0.62	1.65 ± 0.38^▲^
LDL-C, mmol/L	3.07 ± 0.66	2.56 ± 0.52^*∗*^	3.18 ± 0.75	2.58 ± 0.58^▲^
BMI, kg/m^2^	25.86 ± 2.45	25.64 ± 2.75	26.02 ± 3.47	24.65 ± 3.13^▲^
CIMT, mm	1.23 ± 0.46	1.49 ± 0.54^*∗*^	1.24 ± 0.52	1.28 ± 0.41^#^
ΔCIMT, mm	0.22 ± 0.07		0.05 ± 0.02^#^	

^*∗*^ *P* < 0.05, versus the control group before treatment; ^#^ *P* < 0.05, versus the control group after treatment; ^▲^ *P* < 0.05, versus the acarbose group before treatment.
